# Hybrid Treatment of Complex Aortic Arch Anomaly with Saccular Aneurysm

**DOI:** 10.21470/1678-9741-2018-0229

**Published:** 2019

**Authors:** Mustafa Akbulut, Adnan Ak, Kenan Ozturker, Mesut Sismanoglu, Mehmet Altuğ Tunçer

**Affiliations:** 1 Department of Cardiovascular Surgery, Kosuyolu Kartal Heart Training and Research Hospital, Istanbul, Turkey.

**Keywords:** Aorta, Thoracic, Cardiovascular Abnormalities, Subclavian Artery, Aortic Arch Syndromes - Surgery, Dilatation

## Abstract

Aortic arch anomalies are not clinically important unless they cause compression symptoms due to aneurysmatic dilatation. Aortic anomalies need to be treated when they cause complex thoracic aortic diseases, and the treatment approach has evolved over time from open surgical methods, which have high mortality and morbidity rates, to hybrid methods. A case of a 68-year-old male patient with complex aortic arch anomaly treated with hybrid arch repair is reported in this study. Aortic branches were common carotid trunk and aberrant right subclavian artery with a saccular aneurysm.

**Table:** 

Abbreviations, acronyms & symbols	
ARSA	= Aberrant right subclavian artery
CT-A	= Computerized tomography angiography
TEVAR	= Thoracic endovascular stent-graft replacement

## INTRODUCTION

The aortic arch gives rise to three classical branches, the brachiocephalic trunk, the left common carotid artery and the left subclavian artery. In approximately 25% of the population there are many different variations present with no clinical importance unless they refer with compression symptoms. A left aortic arch with an aberrant right subclavian artery (ARSA) is a rare variation of the aortic arch in about 0.5% of the general population^[1]^. In up to 20-30% of these cases, an associated common carotid trunk can be found^[2,3]^.

The classification described by Klieffer indicates in which cases the ARSA should be treated and ARSA is referred to complex thoracic aortic diseases as class 4^[4]^. Aortic arch anomalies have predisposition to aneurysmatic dilatation by sliding out of the normal formation, as in Kommeral diverticulum. Treatment options for ARSA accompanied by complex thoracic aortic diseases include aortic replacement or thoracic endovascular stent-graft replacement (TEVAR), and hybrid approaches with extra-anatomic bypasses. Because of high mortality and morbidity rates of open surgical repair in patients with high perioperative risk, hybrid methods are the most effective and reasonable choice. In our case report, a patient who had a complex aortic arch anomaly and saccular aneurysm in aortic arch was treated with hybrid approach.

## CASE REPORT

**Fig. 1:**
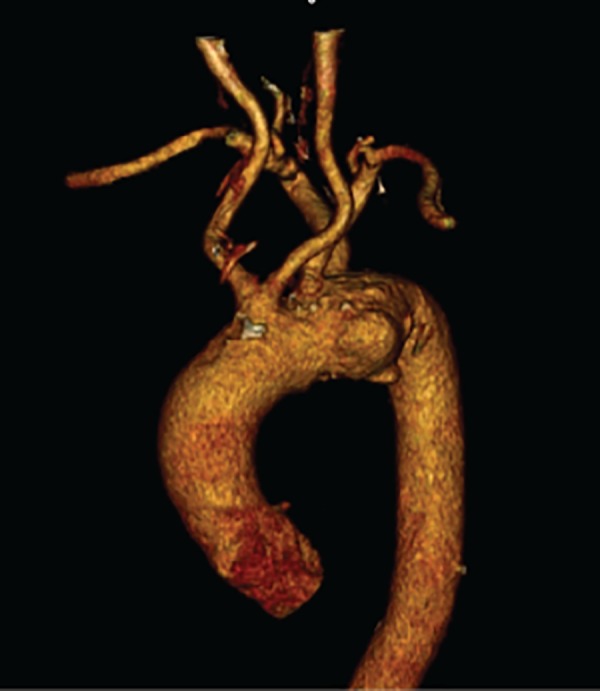
Preoperative CT-A 3D reconstruction

A 68-year-old male patient with hypertension history was admitted to our clinic with chest and upper back pain. In computerized tomography angiography (CT-A) scan, both carotid arteries originated together as a common carotid trunk (Figure 1). The second supra-aortic branch was the left subclavian artery and the third one was the aberrant right subclavian artery. Ascending aorta was normal in diameter (34mm) but saccular aneurysms were detected in small curvature of aortic arch just across the ARSA ostium. A cranial tomography was performed to examine the status of both cerebral circulation and vertebral arteries. It was seen that both vertebral arteries had same diameter and connected to each other to provide posterior cerebral circulation in the circle of Willis. Echocardiography ejection fraction was normal (65%) without any valve diseases. In coronary angiography and carotid Doppler ultrasonography, no evidence of arterial stenosis was found. Serum creatinine levels were normal and there was no history of cerebrovascular event. First stage of the hybrid intervention was performed as bilateral carotico-subclavian bypasses using 7 no Dacron grafts and 5/0 prolen sutures through supraclavicular incisions under general anaesthesia. After the bypasses were finished, proximal left subclavian artery was ligated to the left vertebral artery, but right vertebral artery was too deep to ligate in open surgery.

Following the extra-anatomic bypasses, the patient was transported to catheter lab and right subclavian artery was occluded with a vascular plug (Amplatzer), which was implanted proximal to the right vertebral artery through a right femoral cut down. Subsequently, the saccular aneurysm was repaired by placing a thoracic endovascular stent graft (Bolton Medical (Sunrise, FL), Relay® Plus 46x46x150) to the distal of carotid trunk to create a 2 cm of suitable proximal landing zone.

**Fig. 2:**
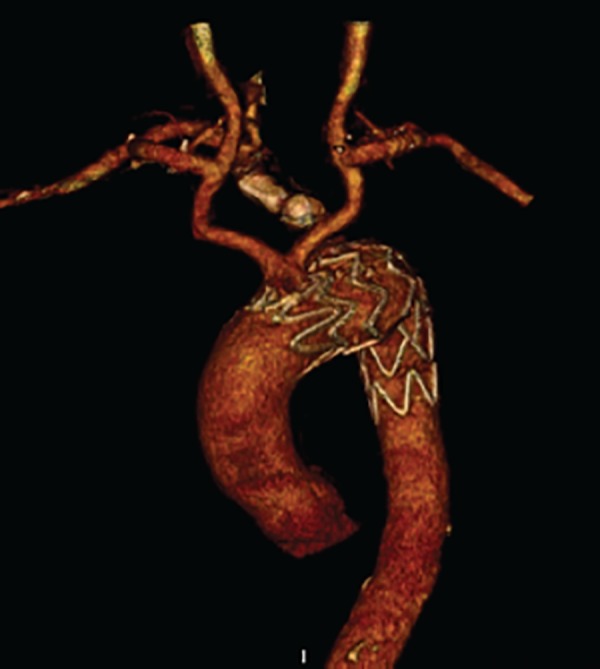
Six Month after operation CT-A 3D reconstruction

The patient was extubated 4 hours after TEVAR and observed for one day in intensive care unit. CT-A scan was performed on the 4^th^ postoperative day and both bypass grafts were patent and no endoleaks were observed in the aneurysm sac. Without any postoperative complications and clinical problems, the patient was discharged and had no problems in both imaging and the 6-month follow-up. (Figure 2)

## DISCUSSION

In the current era of endovascular treatment, hybrid repair methods strengthen surgeons’ arsenal in the treatment of complex aortic diseases and offer simple and safe solutions with less mortality and morbidity in complicated cases compared to open surgery. Kieffer et al.^[4]^ classified the configurations of the accompanying pathologies with ARSA and described in relation to complex thoracic aortic diseases as type 4. In our case, a complicated arch repair is required because of the presence of saccular aneurysms on the opposite aortic wall of right and left subclavian artery ostiums in addition to arch anomaly with ARSA.

Yang modified the Kieffer classification and divided the type of complex aortic diseases into two as type 4a for aneurysm and type 4b for dissection. Yang reported that mortality and morbidity rates of type 4 were higher than those of other types when open surgical repair was performed, however, hybrid interventions reduced these rates^[5]^.

If a 69-year-old patient with no additional disease other than hypertension and a low surgical risk is considered to undergo open surgical repair with median sternotomy, the difficulty of exploration of ARSA advancing behind the oesophagus and the common rooting of common carotid arteries will necessitate a more complicated perfusion strategy which will be challenging for the surgeon. Since the patient's anatomy was suitable for bilateral carotid-subclavian bypass procedure to create a safe landing zone for TEVAR, the hybrid arch repair to avoid cardiopulmonary bypass complications was preferred as well as the need for antegrade cerebral perfusion in our case.

As the left subclavian artery coverage in the same thoracic repair without revascularization, it is a debate in ARSA and most of the surgeons advocate that revascularization of ARSA is physiological and reduces the risk of developing neurological events^[6]^. In our clinic, we examine the posterior cerebral circulation and the dominance in vertebral arteries with cranial CT-A and then make the decision. In our case, both vertebral arteries had the same size and circle of Willis was intact. Occlusions were made proximally in order to make sure vertebral arteries were patent.

In endovascular interventions, most undesired complications are endoleaks. If revascularizations of subclavian arteries are done, subclavian arteries have to be ligated surgically or occluded percutaneously to prevent type 2 endoleaks^[7]^. If ARSA is to be occluded via vascular plug occlusion should be done as close as possible to vertebral artery to prevent erosion or fistulation to the oesophagus^[8]^. In our case left subclavian artery was ligated surgically but the course of the right vertebral artery did not allow to ligate proximally and ARSA was occluded via vascular plug.

## CONCLUSION

The choice of the treatment should be individualized by evaluating the anatomy of aortic arch variations, etiology of disease, diseased zones and comorbidities of the patient. But in the presence of complex aortic diseases hybrid methods should be the first choice of treatment in suitable patients because of their less invasive nature.

**Table:** 

Authors' roles & responsibilities	
MA	Substantial contributions to the conception or design of the work; or the acquisition, analysis, or interpretation of data for the work; final approval of the version to be published
AA	Substantial contributions to the conception or design of the work; or the acquisition, analysis, or interpretation of data for the work; final approval of the version to be published
KO	Substantial contributions to the conception or design of the work; or the acquisition, analysis, or interpretation of data for the work; final approval of the version to be published
MS	Substantial contributions to the conception or design of the work; or the acquisition, analysis, or interpretation of data for the work; final approval of the version to be published
MAT	Substantial contributions to the conception or design of the work; or the acquisition, analysis, or interpretation of data for the work; final approval of the version to be published
